# Letter from B. F. Lightfoot, M. D., Murrayville, Ill.

**Published:** 1868-09-01

**Authors:** B. F. Lightfoot

**Affiliations:** Murrayville, Ill.


*To the Editor of the Chicago Medical Journal :*

Dear Sir : Was called, May 16th, to see a son of Mr. Mc., aged 16 years. Found the patient suffering from what is called colic. I learned from him that he had for a considerable time been suffering from an attack of pain about the umbilicus which came on in the afternoon. He had been visited by Dr. G. of this place previous to my seeing him. He had given him *Podophyllin* as a cathartic, which acted very severely, producing watery discharges. Upon examination I found the abdomen very much swollen, the tumor occupying the centre of the abdomen, and extending into the right iliac region. There was no pain nor tenderness upon pressure; tongue coated white; pulse 120; surface dry and hot. Prescribed *Hydrargyri, Chloridi. Mit*, gr. 60; *Dover Powder*, gr. 36. Divide into six powders, one to be given every four hours. *Potassa Acetas*, ℥ ss ; *Aqua*, ℥ vii. Dose teaspoonful every two hours. May 16th, but little alteration; bowels had moved three or four times, pulse the same. Continued same treatment, with a diminution of the *Hydrarg.* May 17th, tongue beginning to clean off; pulse 110; bowels had moved twice, during which he had passed twenty worms; surface slightly moist, but as yet there is no diminution of the tumor, neither does he manifest any pain or soreness. Ordered *Ferri et Quiniæ Citratis* in doses of six gr. every four hours. May 18th, called in Dr. S. in consultation. Continued same treatment in addition to *Potass. Iodidi.* Continued this treatment for several days with no perceivable change or diminution of the tumor. But the patient gradually gained strength, and during the time walked about the yard. And from this time he began to emaciate gradually, and June 16th was worse again. Tumor was quite as prominent as before, but has become general over the whole abdomen, with considerable pain; pulse 120 per minute with slight vomiting; bowels had moved in the morning, and urine had passed off freely. Prescribed *Opiates*, with *Syrupus Ferri Iodidi.* At this point a pustular eruption made its appearance upon the face, and gradually extended over the whole body. Two days later his bowels began to move off freely, accompanied with much pain. The treatment here consisted of *Astringents*, with *Tonics.* From this time he began to emaciate, and expired on the 15th of July.

A few days previous to his death the tumor became much softer, simulating a dropsical effusion. After the tumor began to soften he passed urine very freely, almost constantly. A *post mortem* examination was requested, but the friends would not consent.

Now to review the case briefly:—1st. The tumor appeared in the form of a pyramid, the umbilicus occupying the top and centre of the pyramid. 2nd. The tumor was as hard as adamant. 3rd. A few days previous to the patient's death the tumor became soft, (and I will state here that no pus was passed at all, neither was there any fluctuation to be felt in the tumor at any time.) His appetite remained good throughout his sickness. He was a mere skeleton at his death. 4th. What was the difficulty?

The case has been reported substantially as it occurred, and if deemed worthy of publication, we should be pleased to have the views of some of the numerous readers of the JOURNAL.

B. F. LIGHTFOOT, M.D.

MURRAYVILLE, ILL., *July* 21*st*, 1868.

